# Pneumonia and influenza, and respiratory and circulatory hospital admissions in Belgium: a retrospective database study

**DOI:** 10.1186/2049-3258-72-33

**Published:** 2014-09-14

**Authors:** Ralph Crott, Isabelle Pouplier, Isabelle Roch, Yi-Chen Chen, Marie-Christine Closon

**Affiliations:** 1 Research Institute of Health and Society (IRSS), Catholic University of Louvain, Clos Chapelle aux Champs 30 bte 3013, Brussels 1200, Belgium; 2 GlaxoSmithKline Vaccines, Avenue Fleming 20, 1300 Wavre, Belgium; 3 Janssen Pharmaceuticals, Singapore, Republic of Singapore

**Keywords:** Hospital admission, Influenza, Pneumonia, Costs, Complication

## Abstract

**Background:**

Influenza infections can lead to viral pneumonia, upper respiratory tract infection or facilitate co-infection by other pathogens. Influenza is associated with the exacerbation of chronic conditions like diabetes and cardiovascular disease and consequently, these result in acute hospitalizations. This study estimated the number, proportions and costs from a payer perspective of hospital admissions related to severe acute respiratory infections.

**Methods:**

We analyzed retrospectively, a database of all acute inpatient stays from a non-random sample of eleven hospitals using the Belgian Minimal Hospital Summary Data. Codes from the International Classification of Diseases, Ninth Revision, Clinical Modification was used to identify and diagnose cases of pneumonia and influenza (PI), respiratory and circulatory (RC), and the related complications.

**Results:**

During 2002–2007, we estimated relative hospital admission rates of 1.69% (20960/1237517) and 21.79% (269634/1237517) due to primary PI and RC, respectively. The highest numbers of hospital admissions with primary diagnosis as PI were reported for the elderly patient group (n = 10184) followed by for children below five years of age (n = 3451).

Of the total primary PI and RC hospital admissions, 56.14% (11768/20960) and 63.48% (171172/269634) of cases had at least one possible influenza-related complication with the highest incidence of complications reported for the elderly patient group. Overall mortality rate in patients with PI and RC were 9.25% (1938/20960) and 5.51% (14859/269634), respectively. Average lengths of hospital stay for PI was 11.6 ± 12.3 days whereas for RC it was 9.1 ± 12.7 days. Annual average costs were 20.2 and 274.6 million Euros for PI and RC hospitalizations. Average cost per hospitalization for PI and RC were 5779 and 6111 Euros (2007), respectively. These costs increased with the presence of complications (PI: 7159, RC: 7549 Euros).

**Conclusion:**

The clinical and economic burden of primary influenza hospitalizations in Belgium is substantial. The elderly patient group together with children aged <18 years were attributed with the majority of all primary PI and RC hospitalizations.

**Trial registration:**

Not applicable.

## Background

Influenza, caused by a contagious respiratory virus, is a common upper respiratory tract illness that results in substantial morbidity and mortality worldwide. Additionally, influenza results in high health care costs and places significant clinical and economic burden on the patient and society [1]. Uncomplicated influenza is characterized by non-specific respiratory symptoms among other general conditions (fever, myalgia, headache, non-productive cough, sore throat and rhinitis) which generally resolve after 3 - 7 days. It is well-known that influenza directly causes viral pneumonia, secondary bacterial pneumonia, upper respiratory tract infections such as sinusitis and otitis media; or facilitates co-infection with other viral and bacterial respiratory pathogens [1,2]. Infection with influenza virus is also associated with the exacerbation of underlying chronic conditions like diabetes, pulmonary (asthma and chronic obstructive pulmonary disorder) and cardiovascular diseases. Evidence suggests that influenza-related complications occur more frequently and are more severe in groups that are at a higher risk of respiratory disease, metabolic disorders and cardiovascular diseases, resulting in high resource utilization and mortality rates. Besides common complications related to influenza, young children are also susceptible to additional non-respiratory tract complications. In some circumstances, infected individuals do not show any symptoms and remain subclinical [1]. Although relatively rare, hospitalization for both respiratory tract and non-respiratory influenza-associated complications could be expensive. There is increasing evidence that influenza infection is not only associated with severe acute respiratory infection such as pneumonia but that it also exacerbates in a number of cases with preexisting chronic diseases, notably cardiovascular ones [3-5]. Influenza-associated complication rates are however influenced by a variety of factors and are notoriously prone to underreporting [6-8].

In Belgium as in most other developed countries the burden of disease and impact on health care systems can be divided into several components which can be presented as a pyramid. At the base of the pyramid are asymptomatic individuals who do not seek health care. Moving bottom-up are individuals with symptomatic uncomplicated cases of influenza who are likely to stay a few days out of work or school since they are unable to perform their daily activities. However, a small proportion of these individuals seek medical ambulatory care, generally through a general practitioner (GP) who in some instances may order a laboratory confirmation test. It is widely established that with the development of influenza-related complications subsequent to infection with the virus, detection of the virus is not usually possible due to its action in indirectly triggering or facilitating other diseases. Moreover, there is no satisfying rapid point-of-care test with high accuracy available for detection of influenza. Consequently, it becomes difficult to estimate the burden of disease of influenza. For complicated cases, when pneumonia is present or suspected, the GP may either refer the patient to a hospital or the patient may visit a hospital emergency department where the patient may be hospitalized for further treatment. Patients and GP’s are free to choose the hospital of their choice in the whole country as there is no restriction in the regional access of care in Belgium.

Accurate estimates of the burden of diseases associated with influenza are therefore important in designing prevention and control measures in both healthy individuals and at-risk groups. We report here findings from a retrospective analysis of data on pneumonia and influenza (PI) and respiratory and circulatory (RC) hospital admissions from the payer’s perspective in Belgium for the time period of 2002–2007. The precise estimate of the attributable fraction of influenza hospitalization is outside the scope of this study.

## Methods

### Data sources

The School of Public Health of the University of Louvain routinely collects data on classical inpatient stays from hospitals that participate voluntarily each year. The hospitals included in the analysis constitute a non-random convenience sample and are distributed across different regions of Belgium (Brussels [n = 2], Flanders [n = 1] Wallonia [n = 8]). Of these eleven hospitals, three are academic hospitals and the remaining hospitals are non-academic.

Data on inpatient stays are based on the official Belgian Minimum Hospital Summary Data (BMHSD) definition of hospital discharge. Hospital stay of each patient is described in the BMHSD set (called “RCM” until 2007) which is recorded for the whole stay after the patient has been discharged. These discharge records are sent to the Ministry of Health twice each year (i.e. June and December). Discharge records for inpatient stays (i.e. when the patient has spent at least one night at the hospital) documenting year of discharge between 2002 and 2007 were included in the study. Therefore, hospital records of patients admitted through the emergency departments and who were discharged within 24 hours from that department were not included in the present analysis.

### Hospital discharge diagnoses

#### Definition of primary main diagnosis

For each patient stay, the BMHSD set contains data on the primary diagnosis and several secondary diagnoses coded as per the International Classification of Diseases, Ninth Revision, Clinical Modification (ICD9_CM) codes. The primary diagnosis was defined as the main reason for hospitalization of the patient for the total duration of hospital stay. Primary diagnosis codes were as follows: PI - ICD9_CM codes 480 - 487 included, and RC - 460 - 519 and 390 - 459.

In the database there are up to maximum 29 different secondary ICD9 codes recorded for each stay. The ICD9-CM codes classification version used by the Belgian hospitals did not vary during the period under study.

#### Definition of other diagnoses and influenza-related complications

The secondary diagnoses describe complications of primary diagnosis and other concomitant pathologies that may have been present or needed to be cared for during the hospital stay (Table 1) [9-15]. A list of complications for all patients was used to diagnose possible influenza-related complications among the secondary diagnoses fields of each hospital admission record, irrespective of the age group for all patients. For children, an additional list of child-specific potential complications was created that was combined with the above “all patients” list and corrected for overlapping ICD9 codes (Tables 1 and 2).

**Table 1:** Code Definition of influenza-related complications for all patients

**Influenza-related complications for all patients**	**ICD-9 codes**
Asthma	493, V17.5
Cardiovascular disease	989.1, 402.01, 402.11, 402.91, 404.01, 404.03, 404.11, 404.13, 404.91, 404.93, 428, 413, 412, 410, 411, 414
Chronic obstructive pulmonary disorder	490, 491, 492, 496
Diabetes	250.xx, 249.xx, V18.0, V77.1, 253.5, 588.1
Hypertension	401-405, 997.91, 459.3x (except codes of the 402 and 404 chapters, already listed under cardiovascular disease)
Pneumococcal pneumonia	481
Secondary respiratory infection	480, 482, 483, 484, 485, 486, 460, 462, 466, 390, 391, 392, 041, 465.9, 034.0, 038.0, 320.2, 482.31
Stroke	430-438, 342

Source: International Classification of Disease – 9^th ^Edition; Codes were selected after a review of the literature [9-15].

**Table 2:** Additional influenza-related complication ICD9 codes in children

**Category**	**Type**	**ICD-9 code**
1° Ear, nose and throat	Acute otitis media	381-381.03, 381.4, 382-382.02, 382.4, 382.9
	Other upper acute respiratory infections	
	- Nasopharyngitis	460
	- Pharyngitis	462
	- Tonsillitis	463
	- Laryngitis and tracheitis	464-464.4
	Acute sinusitis	461-461.9
	Upper respiratory infection	465-465.9
	Influenza with other respiratory manifestations	487.1
2° Lung disease	Acute bronchitis and bronchiolitis	466-466.1, 466.19
		480, 480.8, 480.9, 481, 482,
	Acute pneumonia and influenza	483, 483.8, 485, 486, 487.0
	Asthma	493-493.9
3°Cardiac disease	Pericarditis	420, 420.9-420.99
	Myocarditis	422, 422.9-422.92, 422.99
	Heart failure	428-428.9
4° Neurological Ailments	Reye’s syndrome	331.81
	Encephalopathy	348.3, 487.8
	Febrile convulsions	780.31
	Ataxia	781.3
	Guillain-Barre syndrome	357
5° Renal disease	Glomerulonephritis Nephrotic syndrome, renal failure, Pyelonephritis	580-580.9, 581-581.9, 583-583.7, 583.9, 584-584.9, 586, 590.1-590.9
6° Muscular disease	Myositis and Myoglobinuria	728.0, 729.1, 791.3

Source: International Classification of Disease – 9^th^ Edition; Codes were selected after a review of the literature [11].

### Costs associated with influenza-related hospital admissions

The cost per hospital admission was based on the hospital discharge records from inpatient stays with 2007 as the discharge year. For each hospitalization, the hospitals send a detailed invoice based on the national reimbursement list including medical procedures to the patient’s insurer. Therefore, the costs considered in this study are reimbursement costs. Records for only those patients who are covered by the Belgian National Health insurance (approx. 98% Belgian population) were available in the database and were therefore considered in the cost analyses. The cost per hospital admission included five components – hospital stay charges, pharmacy, laboratory, intensive care and medical procedures and others.

### Statistical analyses

The hospital discharge data was aggregated by month based on the month of admission. Absolute numbers and rates of hospital admissions were estimated by primary diagnosis type (PI and RC), complications and age groups. Five patient groups considered in the present analysis were – infants and toddlers (below 5 years of age), children and adolescents (ages 5 - 17 years), young adults (ages 18 - 49 years), mature adults (ages 50 - 64 years) and the elderly (ages 65 years and above). For mortality data and referrals from nursing home in the elderly the proportion of cases was also estimated. Average length of hospital stay, average annual costs and average costs per hospital admission (Euros) by primary diagnosis type, complications and age groups were also estimated. All data preparation and analysis were performed using *SAS/STAT*® v9.2 or Microsoft *Excel®* software.

## Results

### Hospital admissions by primary discharge diagnosis and complications

**Figure 1:**
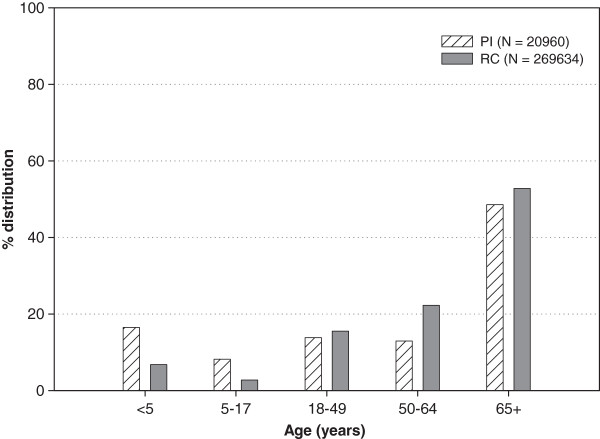
**Age-distribution of primary pneumonia and influenza (PI) and respiratory and circulatory (RC) hospital admissions in Belgium, 2002–2007.** Note: PI – Pneumonia and Influenza, RC - Respiratory and Circulatory.

The total number of hospital admissions due to any cause in the eleven hospitals observed from the years 2002–2007 was 1237517; of these, 1.69% (20960/1237517) and 21.79% (269634/1237517) were classified as primary PI and RC, respectively (PI = 3493 per year (range 3209-3859) RC = 44939 per year (range: 44470-45102). The highest rate of PI and RC hospitalizations were reported in the elderly patient group followed by children below five years of age (Table 3/Figure 1). The burden of hospitalization due to PI and RC was the greatest in the elderly patient group followed by in children aged <18 years. (Table 3/Figure 1). Annual numbers of primary PI and RC hospital admissions (including with and without complications) were similar over the years (data not shown). Of the total primary PI and RC hospital admissions, 56.14% (11,768/20,960) and 63.48% (171172/269634) had at least one influenza-related complication. Complications were most frequently observed in the elderly patient group for both the primary groups, PI and RC (Table 3).

**Table 3:** **Influenza-related hospital admissions and length of stay by age-groups, primary diagnosis and presence of complications in Belgium, 2002**–**2007**

**Total number of hospital admissions**	**(N = 1237517)**					
**Primary diagnosis**	**PI (N = 20960)**			**RC (N = 269634)**		
**Parameters**	**All**	**With complications**	**Without complications**	**All**	**With complications**	**Without complications**
	**(N = 20960)**	**(N = 11768)**	**(N = 9192)**	**(N = 269634)**	**(N = 171172)**	**(N = 98462)**
Hospital admissions, age groups n (%)						
*<5 years*	3451 (3.48%)^*α*^	892 (0.90%)^*α*^	2559 (2.58%)^*α*^	18263 (18.42%)^*α*^	5514 (5.56%)^*α*^	12749 (12.86%)^*α*^
*5-17 years*	1717 (2.52%)^*β*^	343 (0.50%)^*β*^	1374 (2.02%)^*β*^	7469 (10.96%)^*β*^	1510 (2.22%)^*β*^	5959 (8.75%)^*β*^
*18-49 years*	2897 (0.72%)^*γ*^	892 (0.22%)^*γ*^	2005 (0.50%)^*γ*^	41794 (10.38%)^*γ*^	13190 (3.27%)^*γ*^	28604 (7.10%)^*γ*^
*50-64 years*	2711 (1.09%)^*δ*^	1755 (0.70%)^*δ*^	956 (0.38%)^*δ*^	59961 (24.02%)^*δ*^	39520 (15.83%)^*δ*^	20441 (8.19%)^*δ*^
*≥65 years*	10184 (2.44%)^*ϵ*^	7866 (1.89%)^*ϵ*^	2298 (0.55%)^*ϵ*^	142147 (34.02%)^*ϵ*^	111438 (26.67%)^*ϵ*^	30709 (7.35%)^*ϵ*^
Length of stay (mean ± standard deviation) (days)						
- Overall	11.6 ± 12.3	14.6 ± 14.4	7.8 ± 7.4	9.1 ± 12.7	11.3 ± 14.7	5.1 ± 6.4
- By age groups						
*<5 years*^*α*^	5.1 ± 4.9	5.9 ± 7.6	4.9 ± 3.5	4.7 ± 5.8	5.9 ± 7.9	4.2 ± 4.4
*5-17 years*^*β*^	5.3 ± 4.4	6.2 ± 5.0	5.0 ± 4.2	4.1 ± 5.5	6.1 ± 9.0	3.6 ± 4.1
*18-49 years*^*γ*^	8.8 ± 9.8	12.2 ± 14.0	7.3 ± 6.7	5.5 ± 9.6	9.0 ± 14.9	3.9 ± 4.8
*50-64 years*^*δ*^	12.5 ± 13.0	14.0 ± 14.8	9.8 ± 8.3	7.8 ± 12.5	9.4 ± 14.5	4.8 ± 5.9
*≥65 years*^*ϵ*^	15.4 ± 13.8	16.3 ± 14.6	12.3 ± 9.7	11.4 ± 14.0	12.6 ± 14.9	7.2 ± 8.3

Note: % (age-specific proportion) is defined as the relative proportion of hospital admission in the respective age group; age-specific relative proportions and length of hospital stay are calculated on ^α, β, γ, δ, ϵ^which indicate total number of hospital admissions in the respective age group; ^α^99125, ^β^68138, ^γ^402779, ^δ^249585, ^ϵ^417890; PI – pneumonia and influenza; RC – respiratory and circulatory.

The overall mortality rate in patients with primary diagnosis PI and RC were 9.25% (1938/20960) and 5.51% (14859/269634), respectively. Among PI and RC hospital admissions that resulted in death, 4.07% (PI) and 32.16% (RC) had at least one related-complication. The mortality rate was approximately two-fold in patients with complications for primary PI hospital admissions compared to those who developed complications in primary RC hospital admissions (12.12% [1426/11768] versus 6.59% [11279/171172]).

### Length of hospital stay by primary discharge diagnosis and Complications

The average length of hospital stay in patients with primary diagnosis of PI and RC were 11.6 ± 12.3 days and 9.1 ± 12.7 days, respectively. The average length of hospital stay in patients with influenza-related complications was observed to be higher than in the patients without complications for primary PI hospital admissions (14.6 days vs. 7.8 days) and primary RC hospital admissions (11.3 days vs. 5.1 days). The longest duration of hospital stay was observed in the elderly patient group (at least 65 years) for both primary PI (15.4 ± 13.8 days) and RC (11.4 ± 14.0 days) hospital admissions. (Table 3).

### Costs associated with Influenza-related hospital admissions

The overall average costs per hospital admission and costs per admission by age group for the years 2002–2007 are given in Table 4. The average costs per hospital admission for primary PI and RC were 5779 Euros and 6111 Euros, respectively. The relatively higher cost per hospital admission in the RC group than PI is attributed to frequently RC-associated intensive care and complex medical procedures which contributed to a significant proportion of the cost (data not shown).

**Table 4:** **Costs of influenza-related hospital admissions by primary diagnosis and complications in Belgium, 2002**–**2007**

**Average costs per hospital admission (Euros)**	**(N = 1237517)**			
**Primary diagnosis**	**PI (N = 20960)**		**RC (N = 269634)**	
**Parameters**	**All**	**With complications**	**All**	**With complications**
	**(N = 20960)**	**(N = 11768)**	**(N = 269634)**	**(N = 171172)**
- Overall	5779 ± 7072	7159 ± 8353	6111 ± 10341	7549 ± 12068
- By age groups				
*<5 years*	2207 ± 1658	2617 ± 2314	2159 ± 3507	2983 ± 5612
*5-17 years*	2390 ± 1845	2519 ± 2083	2486 ± 5099	3911 ± 9247
*18-49 years*	4493 ± 7464	6777 ± 11922	4404 ± 17120	7655 ± 28737
*50-64 years*	6827 ± 7971	7607 ± 9161	6213 ± 9255	7369 ± 10513
*≥65 years*	7454 ± 7607	7798 ± 8111	7254 ± 8792	7900 ± 9361

Note: PI – pneumonia and influenza; RC – respiratory and circulatory; cost expressed as mean ± standard deviation.

The cost per hospital admission increased with the presence of influenza-related complications for both primary PI and RC hospital admissions (PI: 7159; RC: 7549 Euros). Among patients, the cost of hospital admission was the highest in the elderly group for both PI and RC – 7454 and 7254 Euros per hospital admission, respectively. In both groups, total costs per hospital admission did not increase with the presence of a complication for the elderly patient group (Table 4).

The total cost of PI and RC admissions during 2002–2007 resulting from our sample analysis is estimated to be 121.1 and 1647.7 million Euros, respectively. The total cost of hospitalizations with the presence of a complication contributed significantly to the total cost of all hospital admissions (PI: ~70%, 84.2 million Euros, RC: ~79%, 1292.2 million Euros).

## Discussion

There are very few reports describing the burden related to influenza hospitalizations in Belgium [16]. Between 2002 and 2007, a total of 127906 hospital admissions were due to PI. Nationwide averages of annual hospital admissions with a primary diagnosis were estimated at 1488 (range: 1111 to 1677) for influenza and 24202 (range: 22935 to 25495) for pneumonia [16]. Data from the present retrospective analysis describes the burden of influenza and its related respiratory and non-respiratory complications in hospitals which provide an important public health payer perspective on influenza in Belgium. In our analysis, we estimated the proportions of hospital admissions at 1.69% (n = 20960) and 21.79% (n = 269634) due to primary PI and RC diagnoses respectively during the years 2002–2007. These are in-line with the range reported for Western Europe (0–20%) [17]. Possible influenza-associated complications occurred in 56.14% and 63.48% of primary PI and RC hospital admissions, respectively. The overall mortality rate was estimated at 5.51% and this was higher in the group with a primary PI diagnosis compared to primary RC (9.25% versus 5.51%). A previously published report estimated a relatively higher mortality rate of 11.1% for cases with a primary PI diagnosis for the same time period [16].

The average length of hospital stay was approximately 12 days and 9 days in patients with a primary diagnosis of PI and RC, respectively. These findings were reportedly higher than those observed in Europe recently (average hospital stay ranged from 1.8 to 7.9 days) [17]. The average costs per year were 24.2 and 329.5 million Euros for PI and RC hospital admissions during the study period in our study sample. Complications associated with PI and RC hospitalization were responsible for approximately 70–79% of the total cost of PI and RC hospitalization. Despite the longer duration of stay in patients diagnosed with PI than RC, cost per hospital admission in the primary RC group was relatively much higher. This finding could be suggestive of the fact that RC is frequently associated with intensive care and complex medical procedures when compared to primary PI and therefore the higher hospitalization costs per admission. We estimated an average cost per hospital admission of 5779 Euros due to PI diagnosis. From within the European Union (EU), it is observed that these costs are not comparable. It is previously observed that the average costs in Belgium are much higher than the average hospitalization cost in Germany [18] and in Italy the cost per hospital admission was 3000 Euros which was lower than the average cost reported here [19]. These differences are largely due to the country-specific guidelines on reimbursement resulting from variance in influenza-reporting patterns and differences in healthcare systems across the EU [19].

It is widely suggested that annual vaccination against seasonal influenza remains the most effective way to tackle the burden associated with influenza and pneumonia associated hospitalizations. The World Health Organization (WHO) recommends seasonal influenza vaccination of pregnant women, children aged 6–59 months, elderly individuals (aged ≥65 years) with underlying chronic medical conditions and health care workers. Our data reports high numbers of hospital admissions in young children and the elderly patient group which can, at least partially, be prevented by vaccination [20,21]. As with most countries in the EU, influenza vaccination is recommended and reimbursed in Belgium for people over 65 years or those with chronic diseases, pregnant women and health care workers. In 2008, 46% of the Belgian population at risk, which includes the elderly aged above 65 years or individuals aged above 15 years with a specific chronic disease (asthma, bronchitis, cardiovascular pathology, hypertension renal disease or diabetes) were reported to have been vaccinated during the last influenza season [22]. This modest level of vaccine coverage and the demonstrated burden of influenza underscores the need for a comprehensive approach to controlling its impact in Belgium. A recent cost-effective evaluation conducted for the Belgian setting suggests that universal immunization against influenza may prove to be cost-effective in the prevention and management of influenza [23]. Our findinds collectively with this evidence may help inform immunization recommendations in Belgium. Due to the retrospective nature of our study, we suggest a need for future prospective design patient-level studies to explore the influenza-related burden to society and health economic evaluations to assess the real-life impact-benefit of any programme designed to prevent, control and treat influenza and its associated complications.

An important merit of our study is that a large population from hospitals was investigated across the three regions of Belgium. Additionally, the time period considered in our analysis was six years which is important given the seasonal variation of influenza. Although we report important data here, it is crucial to highlight that it is likely that our findings may have been underestimated. This is due in part attributed to the health care system of Belgium where there are no restrictions in regional access of care in Belgium and the general perception of influenza.

Global comparisons of hospitalization rates associated with influenza are constrained by major difficulties. Hospital admissions for influenza or influenza-related complications are governed by a host of factors that together define the probability of being hospitalized like the severity of symptoms, whether the patient is member of a risk-group, the propensity of the patients to seek health care and the propensity of treating physicians to recommend or initiate a hospitalization. These factors are also influenced by the perceived aggressiveness of the epidemic (epidemic scare) both by the patient and the health care professionals and the overall ease of access (financially and physically) to health care of the local health care system which may have also resulted in underestimation of the incidence of influenza and its related complications.

Besides the inherent limitations of the study database and the inevitable limitations of retrospective design, our analysis presents several limitations. First, our analysis focused for methodological reasons on hospital admissions, and not on discharges. Therefore, the data series are not complete at the end of the period under study (end of 2007) because patients admitted in 2007 and discharged in 2008 were not recorded in the study database which had a cut-off date of 31 December 2007 as discharge year. This estimated loss of information is about 3200 stays, or 1.5% of all the admissions of 2007. Moreover, a large number of patients who died from complications decease at the hospital. However, sometimes the patient may have died outside of the hospital (either at home or in a nursing home generally) based on the type of complication which may also have led to loss of information. Second, given that our hospital sample is a convenience sample and not a random sample, care should be taken to extrapolate the results to the national level i.e. to generalize these findings to all acute hospitals in Belgium since different types of hospitals were included and therefore differences in the outcomes may have existed. Furthermore, this also precluded the calculation of population rates as the population served by the hospitals in our sample (the denominator) was unknown. Third, the number of observed PI admissions that could be attributed to the influenza virus (i.e. the attributable fraction) could not be deduced from the presented data due to lack of laboratory confirmation. Consequently, identification of the exact influenza type could not be ascertained. Identification and estimation of the attributable risk on aggregated data needs more complex statistical methods and additional data that control for confounding variables like other circulating viruses, bacteria and various environmental and meteorological factors [24]. Fourth, regarding the estimations of patients with a non-primary main PI diagnosis but with secondary PI complication, different approaches can and have been used [16,25]. A major problem is the absence in the literature of a commonly agreed set of influenza-related complications. Additionally, one should distinguish between known risk groups for influenza-related complication (say chronic heart failure) and the non-at-risk patients. The BMHSD data allowed only the identification of some of the risk groups, mainly if they are defined by gender, age or discharge diagnosis. In Belgium the different risk groups for a complication of influenza infection are officially defined by a mix of criteria like age (the elderly), living premises (nursing home), various chronic disease statuses or health status (pregnancy), occupation (health care workers) and a combination of age and outpatient therapy. In practice, background information of the patient, including the pre-existence of chronic diseases, is rarely available in the hospital discharge summaries. Consistent with other epidemiological studies our results present aggregated time series data and therefore an ecological bias is always possible. Finally, the database did not have information on vaccination status of the patient and it was therefore difficult to assess if there was a decline in the numbers of influenza-related hospital admissions due to increased vaccination over the years.

## Conclusions

The clinical and economic burden associated with primary influenza hospitalizations and its related complications in Belgium is substantial. Nearly half of the cases of pneumonia and influenza occurred in the elderly both in absolute numbers and in relative proportion of patients with PI. The second largest group was children aged <18 years. Therefore, the group of elderly patients and children are the target group for implementation of prevention and control measures to reduce the morbidity associated with influenza. Further research is needed to prospectively assess influenza-associated hospitalizations using sensitive testing methods in both respiratory and non-respiratory disorders in Belgium.

## Abbreviations

GP: General practitioner; PI: Pneumonia and influenza; RC: Respiratory and circulatory; BMHSD: Belgian Minimal Hospital Summary Data i.e. “Résumé Hospitalier Minimum”; ICD9_CM: International Classification of Disease, Ninth Revision, Clinical Modifications; WHO: World Health Organization.

## Competing interests

Yi-Chen Chen was an employee of GlaxoSmithKline group of companies at the time of the study, and is now employed by Janssen Pharmaceuticals. The institutions of Ralph Crott, Isabelle Roch, Isabelle Pouplier and Marie Christine Closon received grants from GlaxoSmithKline Biologicals SA for conducting the present study. Ralph Crott received consulting fees from GlaxoSmithKline Biologicals SA for other projects.

## Authors’ contributions

RC drafted the manuscript and helped in the design and analysis of the data. IP and IR prepared the data, programmed and performed the descriptive analysis of the data and reviewed the manuscript. M-CC participated in the design, overall coordination and final approval of the manuscript. Y-CC contributed to database analysis design, critically reviewed the manuscript for content. All authors approved the final manuscript.
